# Fetuin-B, a potential link of liver-adipose tissue cross talk during diet-induced weight loss–weight maintenance

**DOI:** 10.1038/s41387-021-00174-z

**Published:** 2021-10-05

**Authors:** Linna Li, Leonard Spranger, Nicole Stobäus, Finja Beer, Anne-Marie Decker, Charlotte Wernicke, Sebastian Brachs, Maria Brachs, Joachim Spranger, Knut Mai

**Affiliations:** 1 Charité – Universitätsmedizin Berlin, corporate member of Freie Universität Berlin, Humboldt-Universität zu Berlin, and Berlin Institute of Health, Department of Endocrinology and Metabolism, 10117 Berlin, Germany; 2 Charité – Universitätsmedizin Berlin, corporate member of Freie Universität Berlin, Humboldt-Universität zu Berlin, and Berlin Institute of Health, Clinical Research Unit, 10117 Berlin, Germany; 3 Charité – Universitätsmedizin Berlin, corporate member of Freie Universität Berlin, Humboldt-Universität zu Berlin, and Berlin Institute of Health, Charité Center for Cardiovascular Research, 10117 Berlin, Germany; 4 DZHK (German Centre for Cardiovascular Research), partner site Berlin, Berlin, Germany

**Keywords:** Obesity, Type 2 diabetes

## Abstract

**Background/objectives:**

Numerous hepatokines are involved in inter-organ cross talk regulating tissue-specific insulin sensitivity. Adipose tissue lipolysis represents a crucial element of adipose insulin sensitivity and is substantially involved in long-term body weight regulation after dietary weight loss. Thus, we aimed to analyze the impact of the hepatokine Fetuin-B in the context of weight loss induced short- and long-term modulation of adipose insulin sensitivity.

**Subjects/methods:**

143 subjects (age > 18; BMI ≥ 27 kg/m^2^) were analyzed before (T-3) and after (T0) a standardized 12-week dietary weight reduction program. Afterward, subjects were randomized to a 12-month lifestyle intervention or a control group. After 12 months (T12) no further intervention was performed until 6 months later (T18) (Maintain-Adults trial). Tissue-specific insulin sensitivity was estimated by HOMA-IR (predominantly liver), ISI_Clamp_ (predominantly skeletal muscle), and free fatty acid suppression during hyperinsulinemic-euglycemic clamp (FFA_Supp_) (predominantly adipose tissue). Fetuin-B was measured at all concomitant time points.

**Results:**

Circulating Fetuin-B levels correlated significantly with estimates of obesity, hepatic steatosis as well as HOMA-IR, ISI_Clamp_, FFA_Supp_ at baseline. Fetuin-B decreased during dietary weight loss (4.2 (3.5–4.9) vs. 3.8 (3.2–4.6) µg/ml; *p* = 2.1 × 10^−5^). This change was associated with concomitant improvement of HOMA-IR (*r* = 0.222; *p* = 0.008) and FFA_Supp_ (*r* = −0.210; *p* = 0.013), suggesting a particular relationship to hepatic and adipose tissue insulin sensitivity. Weight loss induced improvements of insulin resistance were almost completely preserved until months 12 and 18 and most interestingly, the short and long-term improvement of FFA_Supp_ was partially predicted by baseline level of Fetuin-B.

**Conclusions:**

Our data suggest that Fetuin-B might be a potential mediator of liver-adipose cross talk involved in short- and long-term regulation of adipose insulin sensitivity, especially in the context of diet-induced weight changes.

**Trial registration:**

ClinicalTrials.gov number: NCT00850629, https://clinicaltrials.gov/ct2/show/NCT00850629, date of registration: February 25, 2009.

## Introduction

Long-term success of dietary weight loss interventions is known to be limited by frequently observed body weight regain [1, 2]. Recent data indicate that especially adipose tissue function might represent a crucial element in this phenomenon [3]. Identification of molecular pathways regulating adipose function, particularly with respect to adipose insulin sensitivity, may therefore help to unveil the underlying mechanism involved in body weight regulation. Numerous cytokines produced in hepatocytes, myocytes, and adipocytes are involved in the inter-organ cross talk promoting a tissue-specific insulin efficacy [4, 5]. For example, the hepatokine Fetuin-A inhibits insulin receptor tyrosine kinase [6] and is increased in the diabetic and prediabetic state as well as in liver steatosis [7, 8]. It was proposed to induce myocellular insulin resistance in humans [7] via TLR4 mediated adipose tissue inflammation [9].

Fetuin-B, which shares about 22% homology at protein level [10] and belongs to the same family of cysteine protease inhibitors, was recently characterized as a separate hepatokine. It is apparently involved in plaque instability and vascular inflammation [11] and increased levels were found in patients with coronary artery disease, acute coronary syndrome, and acute myocardial infarction [11, 12]. Very recently, elevated hepatic Fetuin-B mRNA expression or circulating Fetuin-B levels were reported in type 2 diabetes [13, 14] and liver steatosis [15–17]. Fetuin-B seems to be particularly associated with intrahepatic lipid content [18], although controversial data were also reported recently [19]. Even if functional similarities between both hepatokines were described, the physiological function seems to be different [10]. The impact of Fetuin-B on insulin sensitivity, which was previously described for Fetuin-A [6, 7, 15], is not entirely clear. Although experimental data indicate a Fetuin-B mediated impairment of insulin action in hepatocytes as well as a decrease of insulin stimulated glucose uptake in myotubes [17], the glucose infusion rate during hyperinsulinemic-euglycemic clamps was not changed by Fetuin-B in mice [17]. Peter and colleagues did not reveal significant correlations between circulating Fetuin-B and any estimate of insulin sensitivity in a cohort of comprehensively phenotyped humans [15]. Nevertheless, a metabolic effect of Fetuin-B was recently supported by numerous data indicating a link to lipid metabolism. An association with elevated total cholesterol, LDL cholesterol, and triacylglycerol was described in some [12, 14] but not in all cohorts [18]. More specifically, knock down of Fetuin-B resulted in increased expression of fatty acid synthase, while CPT1, a key enzyme of lipid oxidation, was suppressed [20]. These data suggest a stimulation of lipolytic pathways by Fetuin-B. However, the exact nature of this relationship remains known. In particular, a potential interaction with insulin-mediated inhibition of lipolysis is still unclear. Insulin-mediated effects on muscular glucose uptake, hepatic gluconeogenesis, and adipose lipid breakdown are strongly modified by body weight reduction [21, 22]. As increased adipose lipid turnover was recently shown to be crucial for sustained weight loss [3], such an effect might be especially relevant in the context of body weight reduction. Therefore, we were particularly interested in the relationship between Fetuin-B and insulin-mediated regulation of lipolysis (free fatty acid (FFA) suppression) during short and long-term course of diet-induced weight loss in humans.

## Materials and methods

### Participants and exclusion criteria

The study was performed between 2010 and 2016 at the endocrine trial center of the Charité Medical School. Screening was performed to rule out abnormal thyroid function and hypercortisolism using 1 mg dexamethasone suppression tests. Moreover, any systemic disease or biochemical evidence of severe hepatic or renal dysfunction was also excluded. Individuals with recent weight changes of more than 5 kg during the last 2 months, with changes of smoking habits or diet behavior during the last 3 months were excluded. Furthermore, patients with severe chronic diseases such as instable coronary heart disease, severe renal insufficiency (eGFR < 30 ml/min), liver diseases, severe psychological diseases, severe endocrine disorders, cancer, chronic infections, or comparable chronic disorders were also excluded. Drugs modifying energy homeostasis and body weight were not allowed during this trial (with exception of thyroxin) [23]. The study protocols were approved by the Institutional Review Board of the Charité Medical School (EA1/140/12). All methods were performed in accordance with the relevant guidelines and regulations. Informed consent was obtained from all participants.

### Study design

Details of the performed weight loss–weight maintenance trial in adults (Maintain-Adult, ClinicalTrials.gov NCT00850629) were already described [23, 24]. In brief, we performed a 12-months randomized controlled weight maintenance intervention in 156 overweight or obese subjects (120 female and 36 male) (BMI ≥ 27 kg/m^2^) after an initial weight loss period of 12 weeks. The major characteristics of the trial are shown in Figure S1. After the initial weight loss period, we compared the effects of a 12-months multimodal lifestyle intervention to maintain body weight with a control group within a randomized controlled trial.

#### Pre-trial weight loss phase

A structured weight reduction program (caloric restriction using a very low energy diet and nutritional counseling, physical exercises, and psychological advices) was used to achieve a weight loss of at least 8%. The detailed protocol was already reported previously [3] and is given in the supplement.

#### Twelve-months randomized weight maintenance phase

Eligible subjects (*n* = 143, 112 female and 31 male) were randomized into the intervention or control group. Subjects in the control group were no longer involved in any form of counseling. A continuous counseling was performed in the intervention group for the next 12 months. This multimodal lifestyle intervention was comparable to sessions of the weight loss period. Details of the protocol and the intervention were already reported [3, 23] and are described in the supplement.

#### Follow-up period

After 12 months all subjects (intervention and control group) underwent a free-living period of 6 months without any further active intervention.

### Randomization and masking

Randomization was performed by the study team using a stratified randomization list. Stratification considered gender and body weight at baseline (three BMI strata). Subjects were not blinded to group assignment.

### Phenotyping

A comprehensive phenotyping was performed before (T-3) and after (T0) weight loss, after 12 months of randomized weight maintenance intervention (T12), and after follow-up of 6 months (T18). All participants received a dietary recommendation of a balanced energy intake for the 3 days preceding phenotyping. The phenotyping focussed on anthropometric, hormonal, and metabolic evaluation. Following a 10-h overnight fast, all patients were investigated at the endocrine trial center of the Charité Medical School at 8.00 a.m. Waist circumference was measured three times and the mean was calculated. At 9.00 a.m. fasting blood samples were taken. Moreover, subjects also underwent a body impedance analysis using AKERN BIA 101 (SMT medical GmbH & Co. KG, Würzburg, Germany) and a hyperinsulinemic-euglycemic clamp at T-3, T0, and T12 as previously described [23, 25]. Blood samples were centrifuged, and plasma and serum samples were frozen immediately at −80 °C.

### Outcomes

The primary outcome defined as weight regain after 18 months (absolute change of BMI from T0 to T18 (kg/m^2^)) was reported previously [23]. Predefined secondary outcomes were the analysis of hormonal, transcriptional, and metabolic predictive markers of body weight regain, metabolic improvement, and cardiovascular risk factors. Currently, we report the data of secondary analyses including estimates of tissue-specific insulin sensitivity and Fetuin-A and B as predictors of long-term changes of adipose insulin sensitivity.

### Laboratory analyses

Capillary blood glucose was measured using the glucose oxidase method (Dr. Müller Super GL, Freital, Germany). Triglycerides, cholesterol, LDL- and HDL-cholesterol, CRP, and liver enzymes were measured by standard laboratory methods using Cobas ISE direct and c111 Analyzer (Roche Diagnostics, Mannheim, Germany) [23]. Serum insulin was measured by fluoroimmunometric assay (AutoDelfia; Perkin Elmer, Rodgau, Germany) (inter-assay CV 2.3–3.5%, intra-assay CV 1.7–2.4%). Non-esterified fatty acids (FFA) were quantified in serum using a commercially available colorimetric assay (NEFA HR2, Wako, Neuss, Germany) performed on ABX Pentra 400 (HORIBA ABX, Montpellier, France) (inter-assay CV < 5%, intra-assay CV < 1%). Fetuin-B was assessed in serum samples by ELISA using a commercial ELISA kit (Human Fetuin B DuoSet ELISA; R&D Systems, Minneapolis, USA), following the manufacturer’s protocol (intra-assay CV: 5.3%, inter-assay CV: 8.2%). Plasma fetuin-A was also determined by an ELISA method (BioVendor Laboratory Medicine, Brno, Czech Republic (intra-assay CV: 3.6%, inter-assay CV: 4.5%)) in accordance with the manufacturer’s instructions.

### Statistics and calculations

Insulin sensitivity was assessed by dividing the average glucose infusion rate (GIR, mg glucose/min) during the steady state of the hyperinsulinemic-euglycemic clamp by the body weight (M-value). The insulin sensitivity index (ISI_Clamp_), which reflects predominantly skeletal muscle insulin sensitivity, was calculated as ratio of M-value to the serum insulin concentration (I, mU/l) in this period of the clamp. HOMA-IR was calculated to assess whole-body insulin sensitivity [26], which is predominantly driven by hepatic insulin efficacy [27]. Effects of insulin on lipolysis were calculated by the suppression of FFA during hyperinsulinemic-euglycemic clamp and expressed as relative changes compared to fasting FFA levels (FFA_Supp_) [28, 29]. Hepatic steatosis index (HSI) was calculated using liver enzymes, BMI, gender, and diabetes state as described by Lee and colleagues [30].

Weight loss-induced changes (T-3 to T0) of specific parameters (Fetuin-A, Fetuin-B, body mass index (BMI), fat mass (FM), waist circumference (WC), HOMA-IR, FFA, FFA_Supp_, ISI_Clamp_, and HSI) were expressed as percentage of baseline values at T-3 (ΔFetuin-A, ΔFetuin-B, ΔBMI, ΔFM, ΔWC, ΔHOMA-IR, ΔFFA, ΔFFA_Supp_, ΔISI_Clamp_, and ΔHSI). Changes of BMI, HOMA-IR, FFA_Supp_, and ISI_Clamp_ between T-3 and T12 were expressed as percentage of baseline values at T-3 (Δ_T3T12_BMI, Δ_T3T12_HOMA-IR, Δ_T3T12_FFA_Supp_, and Δ_T3T12_ISI_Clamp_). Given the negative value of FFA_Supp_, a positive value of ΔFFA_Supp_ or Δ_T3T12_FFA_Supp_ reflect a stronger suppression indicating an improvement of adipose insulin sensitivity.

Statistical procedures were performed using SPSS version 25.0 (SPSS Inc., Chicago, IL, USA) and SAS software, version 9.4 (SAS Institute). Data reported herein reflect secondary analyses and are based on per protocol analysis including data of all available participants at the corresponding time point. Comparisons were made via paired Student’s *t*-test for normally distributed data and Wilcoxon test for skewed data. Correlations between variables were investigated by Pearson’s correlation coefficient for normally distributed data or Spearman’s rank correlation coefficient for skewed data. Data were adjusted for age and gender as described in the “Results” section. Results were considered significant, if the two-sided *α* was below 0.05. Data were presented as median and limits of the interquartile range (IQR: 25th–75th percentile) and plotted as raw values unless stated otherwise.

Multivariate linear regression models were performed to analyze the impact of baseline fasting Fetuin-A or Fetuin-B levels at T-3 on weight loss-induced relative changes of HOMA (ΔHOMA) and FFA_Supp_ (ΔFFA_Supp_). These models included age, gender, and concomitant decline of BMI as potential confounders. Comparable regression models were performed to analyze the impact of baseline Fetuin-B levels on long-term improvement of HOMA and FFA_Supp_. As the hyperinsulinemic-euglycemic clamp was only performed at T-3, T0, and T12, we have chosen these time points for calculation. Given the effect of the 12 months intervention on BMI [23] at T12, the models regarding long-term improvement were also adjusted for treatment group and concomitant BMI changes. Finally, the additive predictive effect of both Fetuin-B on the dependent variables was assessed by likelihood ratio test comparing the models including or excluding Fetuin-B.

Time course of CRP, HSI, liver enzymes, HOMA-IR, ISI_Clamp_, FFA_Supp_, Fetuin-A, and Fetuin-B levels between T-3 and T18 was analyzed using mixed-model, repeated-measures analyses of variance, which considered the correlation between repeated observations and used all available subsequent observations for all participants with values at randomization, regardless of further assessment completion. Means were modeled as a function of study visit (T-3, T0, T12, and T18). The model included adjustment for gender, age, group assignment, and BMI at baseline. An unstructured covariance structure was used. *P* values were adjusted for multiple testing using Bonferroni correction.

## Results

Baseline characteristics of the participants are shown in Table S1 as reported previously [3]. Both, Fetuin-A and Fetuin-B decline with increasing age. Interestingly, Fetuin-B levels were associated with estimates of obesity, liver steatosis (HSI), and insulin resistance (high HOMA-IR, low ISI_Clamp_, and weaker insulin-mediated suppression of FFAs (FFA_Supp_)) (Table 1). Although some of these associations were also found for Fetuin-A, no relationship was seen to estimates of obesity and FFA_Supp_ (Table 1). Given potential effects of age and gender on both fetuins, we repeated these analyses including adjustment for age and gender, which confirmed all relationships (Table S2). To support an independent association of Fetuin-B and FFA_Supp_, an additional adjustment for BMI, HOMA-IR, and ISI_Clamp_ (all known to be associated with FFA_Supp_) was performed. Thereby the relationship between Fetuin-B and FFA_Supp_ was slightly attenuated (*r* = 0.206, *p* = 0.018), but was still observable.

**Table 1:** Association of baseline metabolic and anthropometric parameters with Fetuin-A and B.

Parameter	Fetuin-A [µg/ml]		Fetuin-B [µg/ml]	
	*r*	*p*-value	*r*	*p*-value
Fetuin-B [ng/ml]	0.329	6.5 × 10^−5^		
Age [yr]	−0.221	0.008	−0.166	0.049
BMI [kg/m^2^]	0.125	0.136	0.236	0.005
Fat mass [%]	0.093	0.303	0.266	0.003
Waist circumference [cm]	0.041	0.623	0.085	0.313
HOMA-IR	0.181	0.031	0.220	0.008
ISI_Clamp_ [mg·kg^−1^·min^−1^/(mU·L^−1^)]	−0.168	0.048	−0.247	0.004
FFA [mmol/l]	0.151	0.076	0.229	0.007
FFA_Supp_ [%]	0.103	0.228	0.231	0.006
HSI	0.214	0.011	0.323	1.0 × 10^−4^

While Fetuin-A levels did not differ between females and males (270.4 (240.0–300.0) vs. 236.5 (217.1–29.4) µg/ml; *p* = 0.166), females had higher circulating Fetuin-B levels (4.4 (3.5–4.9) vs. 3.7 (3.2–4.7) µg/ml; *p* = 0.010). However, this gender effect on Fetuin-B disappeared after adjustment to fat mass (data not shown).

### Short and long-term effects of weight loss

**Fig. 1:**
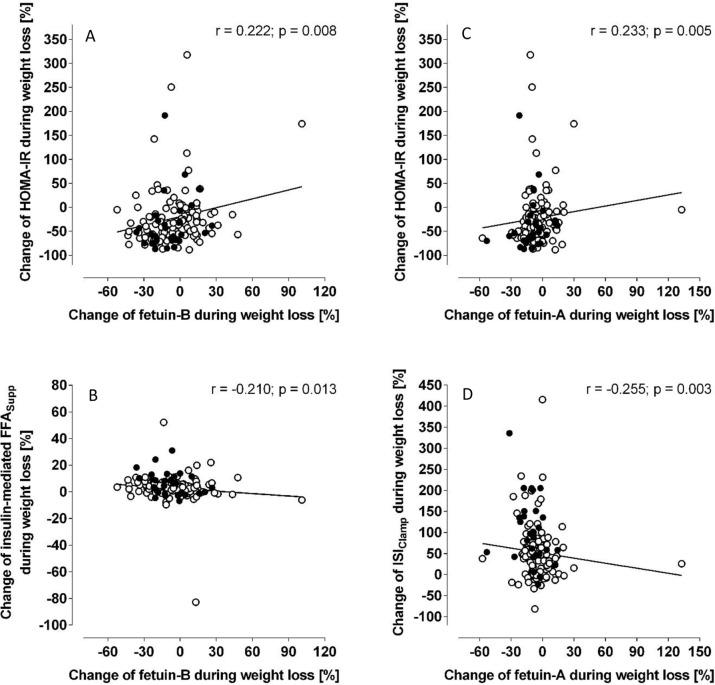
Association of weight loss-induced changes of Fetuin-A and Fetuin-B with reduction of HOMA-IR, insulin sensitivity index (ISI_Clamp_), and insulin-mediated suppression of FFAs (FFA_Supp_) during weight loss. Data were presented for men (filled circle) and women (open circle).

The already reported diet-induced decline of BMI (−4.6 (4.3–4.9) kg/m^2^, Table S3) [24] was accompanied by improvement of estimates of liver steatosis (HSI: 46.5 (42.8–52.5) vs. 40.8 (37.3–46.0); *p* = 1.6 × 10^−20^) and insulin sensitivity (HOMA-IR, ISI_Clamp_ [23] and FFA_Supp_) (Table 2). Fetuin-A as well as Fetuin-B declined during weight loss. The decrease of Fetuin-B correlates with improvement of estimates of obesity (BMI (*r* = 0.337; *p* = 4.2 × 10^−5^), fat mass (*r* = 0.281; *p* = 0.002), waist circumference (*r* = 0.262; *p* = 0.002)), ΔHSI (*r* = 0.179; *p* = 0.034), whole body (ΔHOMA-IR (Fig. 1A)) as well as adipose insulin sensitivity (ΔFFA_Supp_ (Fig. 1B)). No relationship to ΔISI_Clamp_ (*r* = −0.135; *p* = 0.115) was found. Additional adjustment for gender and age did not substantially modify these findings (BMI: *r* = 0.329; *p* = 8.1 × 10^−5^, fat mass: *r* = 0.262; *p* = 0.005, waist circumference: *r* = 0.251; *p* = 0.003, ΔHSI: *r* = 0.166; *p* = 0.051, ΔHOMA-IR: *r* = 0.203; *p* = 0.016, ΔFFA_Supp_: *r* = −0.196; *p* = 0.022, ΔISI_Clamp_: *r* = −0.109; *p* = 0.208). The association of ΔFetuin-B and ΔFFA_Supp_ was still observable after additional adjustment for ΔBMI, ΔHOMA-IR and ΔISI_Clamp_ (*r* = −0.187; *p* = 0.032). This indicates an independent association.

**Table 2:** Fetuin-A and B levels and tissue-specific insulin sensitivity during the trial.

Month	Fetuin-A [µg/ml]	Fetuin-B [µg/ml]	HOMA-IR	ISI_Clamp_ [mg·kg^−1^·min^−1^/(mU·L^−1^)]	FFA_Supp_ [%]
−3	266.0 (158.0–373.9)	4.4 (4.1–4.6)	2.8 (−0.5–6.2)	0.064 (−0.070–0.197)	−89.1 (−90.5– −87.6)
0	249.7 (153.9–345.5)**	4.1 (3.8–4.3)***	1.6 (−1.8–5.0)***	0.089 (−0.154–0.331)***	−92.5 (−93.9– −91.0)***
12	256.3 (154.7–357.3)^#^	4.2 (4.0–4.4)	1.8 (−0.7–4.3)***	0.083 (−0.096–0.262)***	−92.7 (−93.7– −91.7)***
18	256.0 (153.2–358.7)^§^	4.3 (4.0–4.5)	2.3 (−0.3–4.8)*		

Fetuin levels, HOMA-IR, insulin sensitivity index (ISI_Clamp_), and insulin-mediated suppression of FFAs (FFA_Supp_) were reported as estimated marginal means (95% CI) based on mixed-model, repeated-measures analysis of variance adjusted for treatment group, gender, age, and BMI at baseline.

*p < 0.05; **p < 0.01; ***p < 0.001; ^#^*p* = 0.076; ^§^*p* = 0.058 vs. baseline.

In contrast, weight loss induced decline of Fetuin-A was not significantly correlated with the extent of body weight reduction (*r* = 0.156; *p* = 0.063), ΔFFA_Supp_ (*r* = −0.054; *p* = 0.527), ΔWC (*r* = 0.087; *p* = 0.302), ΔFM (*r* = 0.108; *p* = 0.244) and ΔHSI (*r* = 0.128; *p* = 0.130). Only a weak correlation with ΔHOMA-IR and ΔISI_Clamp_ (Fig. 1C and D) could be detected.

After the end of the weight loss intervention, BMI increased between T0 and T18 (1.9 (1.3–2.5) kg/m^2^), Table S3) [23]. Although this regain was accompanied by a moderate impairment of HOMA-IR, ISI_Clamp_, and FFA_Supp_, those parameters were still improved at T12 (HOMA-IR, ISI_Clamp_, and FFA_Supp_) and T18 (HOMA-IR) compared to baseline. In contrast, elevation of Fetuin-A and Fetuin-B between T0 and T18 resulted in fetuin levels at T12 and T18 almost comparable to baseline (Table 2). Accordingly, estimates of liver steatosis (transaminases, HSI) and inflammation (CRP) demonstrated a long-term improvement between baseline and T18 (Table S4). These data were adjusted for baseline BMI, age, gender, and randomization state. Interestingly, long-term improvement of HSI was correlated with the decline of Fetuin-A (*r* = 0.336; *p* = 4.3 × 10^−4^) and Fetuin-B (*r* = 0.216; *p* = 0.027).

### Impact of basal Fetuin-B on short-term modulation of insulin sensitivity

Based on the relationship between HOMA-IR, FFA_Supp_, and Fetuin-B levels at baseline and during weight loss, we wondered if baseline Fetuin-B may be also predictive for weight loss-induced improvement of these estimates of insulin resistance, independent of body weight reduction. To answer this question, we performed a linear regression analysis including age, gender, ΔBMI, and fasting Fetuin-B levels before weight loss as independent variables. While ΔHOMA-IR was not related to basal Fetuin-B levels, a higher ΔFFA_Supp_ was associated with male gender and higher Fetuin-B levels at baseline (Table 3). Although this model explained only about 7% of the ΔFFA_Supp_ during weight loss, a comparable model without Fetuin-B explained a substantially lower variability (*R*^2^ = 0.026; *p* = 0.018 for comparison between models). Furthemore, we additionally adjusted all analyses for ΔFetuin-B, as ΔFetuin-B was associated with basal Fetuin-B levels. This did not modify the results. Notably, basal Fetuin-A was not related to ΔFFA_Supp_ (Table S5)_._

**Table 3:** Prediction of weight loss-induced change of insulin-mediated suppression of FFAs (ΔFFA_Supp_) by baseline Fetuin-B level and gender.

Predictors	Coefficients	95% CI	*p* value	*R*^2^
Fetuin-B before weight loss	1.42	0.25–2.59	0.018	0.066*
Gender (reference: male)	−4.80	−9.10– −0.50	0.029	
ΔBMI	0.16	−0.33–0.64	0.522	
Age	0.02	−0.16–0.12	0.748	

*p = 0.057.

### Impact of basal Fetuin-B on long-term modulation of insulin sensitivity

Given the predictive impact of Fetuin-B on short-term improvement of FFA_Supp_ during weight loss (ΔFFA_Supp_), we finally aimed to analyze, whether baseline Fetuin-B levels can also predict long-term changes of adipose insulin sensitivity. Therefore, we performed comparable linear regression models including age, gender, fasting baseline Fetuin-B levels, randomization group, and concomitant long-term change of BMI. Basal Fetuin-B was again not related to long-term changes of HOMA-IR. However, in addition to male gender and long-term weight changes, higher baseline Fetuin-B levels were associated with Δ_T3T12_FFA_Supp_ (Table 4). Additional adjustment for Δ_T3T12_Fetuin-B did not modify this finding. A comparable model without Fetuin-B explained a substantially lower variability (*R*^2^ = 0.098; *p* = 0.002 for comparison between models) than the full model. In analogy to previous analyses, basal Fetuin-A levels were not predictive for changes of adipose insulin sensitivity (Table S6).

**Table 4:** Prediction of long-term improvement of insulin-mediated suppression of FFAs between T-3 and T12 (Δ_T3T12_FFA_Supp_) by baseline Fetuin-B level, gender, and concomitant change of BMI.

Predictors	Coefficients	95% CI	*p* value	*R*^2^
Fetuin-B before weight loss	1.95	0.72–3.19	0.002	0.178***
Gender (reference: male)	−4.60	−8.33– −0.88	0.016	
Δ_T3T12_BMI	−0.24	−0.43 – −0.04	0.017	
Age	0.00	−0.12–0.13	0.967	
Randomization group	0.45	−2.62–3.52	0.771	

***p = 1.2 × 10^−3^.

## Discussion

Hepatic cytokines have been shown to affect different insulin target tissues. In accordance with the metabolic impact of Fetuin-A, our findings confirmed previously described associations of Fetuin-A with estimates of liver steatosis as well as the impact on whole body and myocellular insulin resistance [7, 8, 31]. Fetuin-B could represent a novel player also involved in the metabolic inter-organ cross talk. In line with such an assumption, we confirmed data revealing a relationship of Fetuin-B to estimates of obesity [15, 18], liver fat [16–18], whole body [14, 17, 18], and myocellular insulin resistance [17], even if this was not described in all cohorts [15, 19]. However, previous findings also demonstrate a correlation of Fetuin-B with parameters of lipid metabolism [12, 14, 20]. This suggests a potential role of Fetuin-B in the regulation of adipose tissue function. As a substantial role of weight loss-induced modification of adipose tissue function on body weight maintenance was revealed recently by us [3], Fetuin-B might be of particular interest for long-term body weight regulation. Now we report for the first time a relationship between Fetuin-B and insulin-dependent suppression of FFAs, an estimate of adipose insulin sensitivity. Given the independent association of high Fetuin-B levels with low insulin-mediated suppression of adipose tissue lipolysis it is tempting to speculate, that Fetuin-B might represent a crucial element diminishing antilipolytic effects of insulin, e.g., during postprandial hyperinsulinemia. The observed association of Fetuin-B with fasting FFAs suggests that Fetuin-B could be also relevant in the regulation of fasting lipolysis. However, this relationship could vice versa also reflect a FFA mediated increase of Fetuin-B release, as numerous polyunsaturated fatty acids can activate farnesoid X receptor (FXR) [32], a nuclear receptor known to induce hepatic Fetuin-B expression [33].

In the context of such an effect of Fetuin-B on lipolysis, elevated Fetuin-B levels in obesity and fatty liver disease might represent a counterbalancing mechanism to attenuate further lipid storage. Accordingly, a weak association of weight loss-induced short- and long-term decline of Fetuin-B and estimates of liver steatosis (HSI) was revealed in our cohort. Interestingly, this assumption is further supported by experimental data. Fetuin-B knockdown in liver cells resulted in increased lipid content and expression level of genes involved in fatty acid synthesis, whereas key enzymes of fatty acid oxidation declined [20].

Our study revealed that increased Fetuin-B level in obesity could be attenuated by dietary weight loss. This would result in a reduction of previously described antilipogenic properties of Fetuin-B. Actually, weight loss-induced decline of Fetuin-B was associated with an increase of insulin-mediated FFA suppression in our trial. This interaction seems to be specific for adipose tissue, as changes of myocellular insulin sensitivity were not related to Fetuin-B reduction.

Although our data mostly reflect associations, it is tempting to speculate that elevated Fetuin-B levels represent a novel mechanism supporting adipose insulin resistance found in subjects with obesity and increased liver fat [34]. Interestingly, higher Fetuin-B levels are apparently also relevant for regulation of adipose tissue function during diet-induced weight loss–weight maintenance, as both short and long-term improvement of adipose insulin sensitivity was independently associated with higher baseline levels of Fetuin-B. Even if the cellular structures potentially underlying such an interaction of Fetuin-B and adipose insulin resistance are currently unknown, this adds relevant novel aspects to existing knowledge. Thus, further research is clearly warranted to elucidate the details and even the direction of the here observed relationship.

Parts of our findings are in contrast to recent data. Although we confirmed previous reports demonstrating a weight loss-induced decline of Fetuin-A [7, 35, 36], the decline of Fetuin-B was modest in most yet analyzed cohorts [15, 19]. Differences of baseline characteristics and especially the lower number of study participants [19] or the degree of weight loss [15] might explain the stronger and significant effects observed in our study. Furthermore, Peter and colleagues demonstrated a relationship of weight loss-induced changes of insulin sensitivity and modification of Fetuin-A but not Fetuin-B [15]. However, these data were primarily based on estimates of global insulin sensitivity during oral glucose load, while we aimed to use estimates of tissue-specific insulin sensitivity. This may provide a different perspective into the interaction of Fetuin-B with insulin action and may explain the different findings.

Nevertheless, the interpretation of our data is limited by some additional factors. First, as most of our data are based on associations, further data are clearly required. However, some experimental and animal findings are in accordance with our results in humans and provide potential molecular explanations [20]. Next, several behavioral, social, and environmental factors are known to have substantial impact on long-term effects of dietary weight loss interventions [37, 38]. Although we aimed to standardize the dietary intake and physical activity during the group sessions, we cannot exclude that these factors may have influenced our results. Moreover, hepatic steatosis was not measured directly, even if the HSI is used in several trials as a surrogate of liver steatosis [30, 39, 40]. Finally, numerous techniques are described to assess adipose tissue insulin sensitivity. Even if hyperinsulinemic-euglycemic clamp is considered as a well-established analytical approach, different insulin infusion rates (4–80 mU/m^2^, partly used stepwise) [29, 41–43] and outcome measures (suppression of plasma FFAs, suppression of plasma glycerol or glycerol and FFA rates of appearance using tracer dilution technique) [29, 41, 43, 44] were used. Interestingly, results based on low dose insulin infusion rates might be influenced by changes of lipolysis in skeletal muscle, which is rather stable during high insulin dosage [43]. These data using tissue-based microdialysis technique indicate that a higher dose of insulin, which results in comparable insulin levels during steady state as seen in our participants, should be preferred to differentiate suppression of lipolysis in skeletal muscle and adipose tissue. Although tracer dilution technique is widely accepted to assess adipose tissue lipolysis, suppression of FFA during hyperinsulinemic-euglycemic clamp is also frequently used [28, 34, 44–46]. Given these data, we believe that we used a valid approach, even if adipose tissue microdialysis was not performed in this study.

On the other hand, numerous strengths of the current trial should be mentioned. These include the large sample size, the long duration of the intervention, subsequent observation, and the comprehensive phenotyping including detailed assessment of myocellular and adipose tissue insulin sensitivity by hyperinsulinemic-euglycemic clamp in such a large cohort.

Taken together, we revealed a novel relationship of Fetuin-B to several markers of insulin resistance, especially estimates of adipose tissue function. If this would reflect a causal relationship, such a mechanism could counterbalance further lipid storage in obesity and fatty liver disease. Weight loss-induced changes of Fetuin-B were primarily associated with an increase of whole body and adipose insulin sensitivity. Most interestingly, basal Fetuin-B level were related to short- and long-term improvement of adipose insulin sensitivity inhibiting lipid breakdown during hyperinsulinemia. Considering previous experimental data, Fetuin-B might be therefore linked to insulin-dependent FFA metabolism in adipose tissue, which highlighted this molecule as a promising target involved in long-term regulation of adipose tissue function and body weight regulation. In any case, our prospective data support that further studies should elucidate the role of Fetuin-B in the context of organ-specific glucose and lipid metabolism.

## Supplementary information

Online Appendix_clean

Online Supplemental Materials_Gcp_protocol

## Data Availability

The datasets generated during and/or analyzed during the current study are available from the corresponding author on reasonable request.
