# Supplementary material for: Fetuin-B, a potential link of liver-adipose tissue cross talk during diet-induced weight loss–weight maintenance
Source: Nutr Diabetes. 2021 Oct 5;11:31. doi: 10.1038/s41387-021-00174-z (PMC8492646; doi:10.1038/s41387-021-00174-z)
Supplement: Supplementary file 1 — Online Appendix_clean [file 41387_2021_174_MOESM1_ESM.docx]

**Supplemental Data**

**Fetuin-B, a potential link of liver-adipose tissue cross talk during diet induced weight loss - weight maintenance**

by

Linna Li^*^, Leonard Spranger^*^, Nicole Stobäus, Finja Beer, Anne-Marie Decker, Charlotte Wernicke, Sebastian Brachs, Maria Brachs, Joachim Spranger, Knut Mai

*Study design.*

*Pre-trial weight loss phase:*

The protocol of the 12 weeks’ weight loss program included three components (1): caloric restriction, nutritional counseling and physical exercises. Caloric restriction was applied during the weight reduction program in two stages. A replacement of all 3 meals by a very-low energy diet (Optifast 2, Nestlé HealthCare Nutrition GmbH, Frankfurt am Main, Germany) was performed during the first eight weeks. This diet supplied 800 kcal per day and was provided by the trial team for free. Participants received 35 portions of formula diet for each week (5 per day). The participants were advised not to consume any additional food. After 8 weeks the diet was switched to an energy-reduced healthy diet composed as a balanced mix (carbohydrates 35-45 %, fat 25-35 %, and protein 25-30 %). Although daily calorie intake of approximately 1500 kcal was recommended, individual counseling was performed based on information of the initial eating protocols, measured energy expenditure and reported physical activity.

Weekly meetings including dietary advices for healthy living, group workshops with practical cooking exercises and recommendation regarding increased physical activity were performed during the entire weight loss period of 12 weeks (1). Psychologists also attended the workshops during four dates at week 5, 7, 9 and 11. A physician gave medical advice during one of the meetings as well. Compliance of the diet was supported by providing specific recipes, cooking advices, and instructions for behavior modifications (only 3 meals per day, at least 4 hours break between the meals, reduced carbohydrate intake at dinner). In the first two months, weight loss and health status were monitored weekly, which included a patient interview and blood pressure measurement. Body weight was measured at least once per week. A supervised 30 min exercise session was performed after each meeting to support the recommended increase of physical activity. Participants were also encouraged to attend at least one additional physical exercise course per week (1).

*12-months randomized weight maintenance phase:*

Subjects who lost at least 8% of their body weight during the weight loss phase (n=143, 112 female and 31 male) were randomized into an intervention and a control group. Subjects in the control group received an advice leaflet and were asked to return for examination after 12 and 18 months. However, they were no longer involved in any form of counseling (1). Continuous counseling was performed in the intervention group for the next 12 months in gradually diminishing frequency. Weekly group sessions, comparable to sessions of the weight loss period, were performed for the first sixteen weeks of the weight maintenance period. Subsequently meetings were taking place each two weeks over a period of two months. Afterwards monthly meetings were performed until the end of the intervention period. Thus, at least 36 meetings were offered every participants of the intervention group during 12 months weight maintenance phase. The dietary advices within the intervention group were focused on a balanced diet advocating the preferential intake of specific foods (like high intake of vegetables, cereals, fat reduced foods, lean meat consumption (lean fish and chicken)). The recommended distribution of macronutrients (35-45% carbohydrates, 25-35% fat, and 25-30% protein) was comparable to the final phase of the weight loss intervention. An individual caloric intake was calculated and further adapted to achieve body weight maintenance. Therefore, body weight was measured during every group session.

The supervised physical activity regime was maintained for the first 12
weeks of weight maintenance period. Thereafter participants were encouraged to exercise at least twice a week but without direct supervision. To increase motivation and allow self-monitoring, pedometers were given to the participants and a gym membership was offered. The mentioned psychological support was continued for 6 additional dates (1).

*Follow-up period:*

After 12 months all subjects (intervention and control group) underwent a free living period of 6 months without any further active intervention.

*Power calculation.*

Weight change was used for power calculation (1). This considered the fact that the variance of any causal endocrine parameter should be smaller than that of body weight, since the latter is also affected by other parameters such as socio-economic or life-style factors, which do not necessarily modify endocrine circuits. We aimed to identify a weight difference of 1.15% between intervention and control group. This reflects 30% of a previously described effect after 6 months (2). Given a variance of 1.96% in the control group, we estimated a sample size of 46 individuals per treatment arm would be required to provide 80% power with an α-error rate of 5% (query 7.0). We assumed a 20% drop out rate during the initial weight loss period and about 15% drop outs during the randomized intervention period. Therefore at least 144 adults had to be included in the weight reduction period (T-3).

**Figure S1.** Trial profile of Maintain-Adults

Flow chart of the randomized controlled trial in adults. f/m indicates female/male.

Drop out (n = 13 (8.3%))

♦ Lost interest (n=5)

♦ Unable to achieve weight loss ≥ 8% (n=6)

♦ Unable to attend group sessions (n=2)

Analyzed after 18 months (n=51) (f 39; m 12)

♦ Not available for follow-up (unable to be contacted) (n=7)

Analyzed after 12 months (n=58) (f 45; m 13)

♦ Not available for follow-up (unable to be contacted) (n=13)

Analyzed after 12 months (n=64) (f 50; m 14)

♦ Not available for follow-up (unable to be contacted) (n=8)

Analyzed after 18 months (n=61) (f 48; m 13)

♦ Not available for follow-up (unable to be contacted) (n=3)

## 12 months randomized intervention

Randomized (n=143)

## Enrollment

Assessed for eligibility (n=223)

Excluded (n= 67)

♦  Not meeting inclusion criteria (n=67)

Inclusion in 12-week weight reduction program (n= 156)

Allocated to intervention group (n=72); (f 56; m 16)

♦ Received allocated intervention (n=72)

Allocated to control group (n=71) (f 56; m 15)

♦ Received allocated intervention (n=71)

## Allocation

## 6 months follow up

**Supplemental tables**

**Table S1: Basal characteristics of the participants.**

Metabolic and anthropometric parameters of the randomized participants before weight loss. Results are presented as median and interquartile range (IQR).

| Parameter | No. of  partici-pants | before weight loss | |
| --- | --- | --- | --- |
|  |  | median | (IQR) |
| Females [n (%)] |  | 112 (78) |  |
| Postmenopausal females [n (%)] |  | 58 (51) |  |
| Age [yr] | 143 | 50.5 | (41.7-60.8) |
| BMI [kg/m^2^] | 143 | 35.6 | (32.9-41.0) |
| Fat mass [%] | 126 | 37.4 | (32.6-40.0) |
| Waist circumference [cm] | 143 | 106.5 | (97.0-117.0) |
| Total cholesterol [mg/dl] | 143 | 200.0 | (176.0-233.0) |
| HDL-cholesterol [mg/dl] | 143 | 49.3 | (40.6-61.3) |
| LDL-cholesterol [mg/dl] | 143 | 123.1 | (103.2-146.8) |
| Triacylglycerol [mg/dl] | 143 | 126.0 | (85.0-169.0) |
| HbA1c [mmol/mol] | 143 | 37.7 | (34.4-42.1) |
| HbA1c [%] | 143 | 5.6 | (5.3-6.0) |
| HOMA-IR | 142 | 2.2 | (1.4-3.4) |
| ISI_Clamp_ [mg•kg^−1^•min^−1^/(mU•l^−1^)] | 139 | 0.06 | (0.04-0.08) |
| FFA [mmol/l] | 140 | 0.65 | (0.51-0.83) |
| Insulin-mediated FFA suppression [%] | 139 | -91.5 | (-85.7-(-94.4)) |
| Insulin concentration during clamp [mU/l] | 139 | 67.5 | (57.4-76.0) |
| AST [U/l] | 141 | 26.0 | (22.0-32.1) |
| ALT [U/l] | 141 | 26.6 | (20.7-36.4) |
| GGT [U/l] | 140 | 25.1 | (18.8-39.5) |
| HSI | 141 | 46.5 | (42.8-52.5) |
| CRP [mg/dl] | 141 | 0.35 | (0.16-0.62) |
| Fetuin-A [µg/ml] | 143 | 268.4 | (226.6-297.7) |
| Fetuin-B [µg/ml] | 142 | 4.2 | (3.5-4.9) |

**Table S2: Association of baseline metabolic and anthropometric parameters with Fetuin-A and B, adjusted for age and gender.**

| Parameter | Fetuin-A [µg/ml] | | Fetuin-B [µg/ml] | |
| --- | --- | --- | --- | --- |
|  | r | p-value | r | p-value |
| Fetuin B [ng/ml] | 0.282 | 0.001 |  |  |
| BMI [kg/m^2^] | 0.110 | 0.195 | 0.239 | 0.004 |
| Fat mass [%] | -0.015 | 0.871 | 0.165 | 0.068 |
| Waist circumference [cm] | 0.142 | 0.093 | 0.221 | 0.009 |
| HOMA-IR | 0.206 | 0.015 | 0.264 | 0.002 |
| ISI_Clamp_ [mg•kg^−1^•min^−1^/(mU•L^−1^)] | -0.220 | 0.010 | -0.314 | 2.0*10^-4^ |
| FFA [mmol/l] | 0.147 | 0.085 | 0.203 | 0.017 |
| FFA_Supp_ [%] | 0.161 | 0.060 | 0.338 | 5.6*10^-5^ |
| HSI | 0.189 | 0.026 | 0.314 | 2.0*10^-4^ |

**Table S3: BMI of the entire group during the trial**

BMI was reported as estimated marginal means (95% CI) based on mixed-model, repeated-measures analysis of variance adjusted for treatment group, gender and age.

| Month | BMI [kg/m^2^] |
| --- | --- |
| -3 | 37.3 (36.2 – 38.2) |
| 0 | 32.6 (31.6 – 33.5)*** |
| 12 | 33.4 (32.4 – 34.4) ***^,§§^ |
| 18 | 34.5 (33.4 – 35.5) ***^,§§§^ |

*** p<0.001 vs. baseline; §§ p<0.01 and §§§ p<0.001 vs. month 0

**Table S4: Estimates of liver function and inflammation during the trial.**

Transaminases, hepatic steatosis index (HSI) and CRP were reported as estimated marginal means (95% CI) based on mixed-model, repeated-measures analysis of variance adjusted for treatment group, gender, age and BMI at baseline.

| Month | AST [U/l] | ALT [U/l] | HSI | CRP [mg/dl] |
| --- | --- | --- | --- | --- |
| -3 | 28.0 (26.6 - 29.3) | 31.5 (28.6 - 34.4) | 47.5 (47.0 - 48.0) | 0.52 (-0.11 - 1.14) |
| 0 | 26.2 (24.6 - 27.8) | 26.9 (22.3 - 31.5) | 42.0 (41.3 - 42.8)*** | 0.42 (-0.21 - 1.04)^#^ |
| 12 | 24.9 (23.7 - 26.1)*** | 24.1 (22.5 - 25.6)*** | 42.6 (41.8 - 43.4)*** | 0.31 (-0.31 - 0.93)*** |
| 18 | 24.0 (23.0 - 25.0)*** | 23.3 (21.7 - 24.8)*** | 43.4 (42.5 - 44.3)*** | 0.35 (-0.27 - 0.97)*** |

*** p<0.001; # p=0.068 vs. baseline

**Table S5: Linear regression model analyzing effects of baseline Fetuin-A, age, gender and ΔBMI on weight loss induced change of insulin mediated suppression of FFAs (ΔFFA_Supp_).**

| Predictors | Coefficients | 95% CI | p value | R^2^ |
| --- | --- | --- | --- | --- |
| Fetuin-A before weight loss | -0.011 | -0.05 – 0.02 | 0.549 | 0.029^#^ |
| Gender (reference: male) | -3.65 | -7.94 – 0.65 | 0.095 |  |
| ΔBMI | 0.12 | -0.37 – 0.61 | 0.629 |  |
| age | -0.07 | -0.21 – 0.07 | 0.341 |  |

#p=0.413

**Table S6: Linear regression model analyzing effects of baseline Fetuin-A, age, gender and gender and concomitant change of BMI on insulin mediated suppression of FFAs between T3 and T12 (Δ_T3T12_FFA_Supp_).**

| Predictors | Coefficients | 95% CI | p value | R^2^ |
| --- | --- | --- | --- | --- |
| Fetuin-A before weight loss | 0.005 | -0.03 – 0.04 | 0.790 | 0.098^#^ |
| gender (reference: male) | -3.29 | -7.09 – 0.51 | 0.089 |  |
| Δ_T3T12_BMI | -0.28 | -0.48 – -0.07 | 0.008 |  |
| age | -0.04 | -0.16 – 0.09 | 0.593 |  |
| randomization group | 0.44 | -2.86 – 3.75 | 0.791 |  |

#p=0.057

**References**

1. Mai K, et al. Effects of a combined dietary, exercise and behavioral intervention and sympathetic system on body weight maintenance after intended weight loss: Results of a randomized controlled trial. *Metabolism*. **83**, 60-7 (2018).

2. Woo J, et al. Effectiveness of a lifestyle modification programme in weight maintenance in obese subjects after cessation of treatment with Orlistat. *J EvalClinPract*. **13(6)**, 853-9 (2007).
