# Supplementary material for: Fetuin-B, a potential link of liver-adipose tissue cross talk during diet-induced weight loss–weight maintenance
Source: Nutr Diabetes. 2021 Oct 5;11:31. doi: 10.1038/s41387-021-00174-z (PMC8492646; doi:10.1038/s41387-021-00174-z)
Supplement: Supplementary file 2 — Online Supplemental Materials_Gcp_protocol [file 41387_2021_174_MOESM2_ESM.pdf]

Hormonal control of body weight maintenance: effects of a randomized controlled lifestyle intervention trial

„MAINTAIN“

ClinicalTrials: NCT00850629

Date 25.02.2009

In the context of the Clinical Research Group (DFG) 218/0: Hormonal regulation of body weight maintenance

Speaker: Prof. Dr. Annette Grüters-Kieslich (Charité, CCM, Charitéplatz 1, 10117 Berlin, Germany)

Coordinators: Prof. Dr. Heiko Krude (Department of Pediatric Endocrinology and Diabetology, Charité Universitätsmedizin Berlin, Germany) and Prof. Joachim Spranger (Department of Endocrinology & Metabolism, Charite – Universitätsmedizin Berlin, Germany)

Contents

1 Synopsis ... 4
2 Summary ... 8
3 Background and Rationale ... 9
4 Overview of Trial Design ... 10
4.1 Specific hypotheses and aims ... 10
4.2 General study design ... 10
4.3 Study Flow ... 12
5 Interventions ... 13
5.1 Pre-trial weight loss phase: ... 13
5.2 12 months randomized weight maintenance phase: ... 14
6 Definition of primary and secondary endpoints ... 15
6.1 Primary endpoints ... 15
6.2 Secondary endpoints ... 15
7 Study Population ... 16
7.1 Inclusion criteria: ... 16
7.2 Exclusion criteria: ... 17
7.3 Recruitment ... 17
7.4 Screening ... 17
8 Measures and Procedures ... 18
8.1 Informed Consent ... 18
8.2 Procedures ... 18
8.2.1 Phenotyping ... 18
8.2.2 Anthropometry ... 19
8.2.3 Bioimpedance measurements to estimate body composition ... 19
8.2.4 3-day nutrition protocol ... 19
8.2.5 Social, Economic and Health Related Questions ... 20
8.2.6 Assessment of physical fitness by measuring aerobic capacity ... 20
8.2.7 Indirect calorimetry ... 20
8.2.8 Oral glucose tolerance test (OGTT) ... 20
8.2.9 Biopsies of adipose and muscle tissue ... 21
8.2.10 Hyperinsulinemic euglycemic clamps ... 21

8.2.11 Cortisone kinetics 21
8.2.12 Saliva 22
8.2.13 24h-urine sampling (cortisol and metanephrines) 22
8.2.14 Intima media thickness 22
8.3 Assessment Schedule 22
8.4 Randomization 23
8.5 Participant Safety 23
9 Data management 23
10 Statistical Considerations 24
10.1 Power analysis and sample size calculation 24
10.2 Data analysis plan 24
11 Appendix 25

1 Synopsis

**Title of the clinical study**	Hormonal regulation of body weight maintenance
**Type of the study**	Randomized controlled clinical trial
**Principal Investigators**	Prof. Dr. Joachim Spranger<br>Clinic of Endocrinology, Diabetes and Nutrition<br>Charité - Universitätsmedizin Berlin<br>Charitéplatz 1 , 10117 Berlin<br>Tel: +49/30/450-514252<br>Email: joachim.spranger@charite.de<br><br>PD. Dr. Knut Mai<br>Clinic of Endocrinology, Diabetes and Nutrition<br>Charité - Universitätsmedizin Berlin<br>Charitéplatz 1 , 10117 Berlin<br>Tel: +49/30/450-514252<br>Email: knut.mai@charite.de
**Objective**	The investigators here propose to perform a collaborative clinical research effort including a randomized controlled trial investigating the mechanisms of weight maintenance and their relation to a lifestyle intervention in adults. The detailed investigation and analysis of the variability and dynamics of the endocrine circuits responding to a negative energy balance and weight loss will be accompanied and enhanced by specific clinical projects targeting peripheral and central-nervous aspects of hormonal counter-regulation after weight loss. Mechanisms of endocrine counter-regulation and potential therapeutic approaches will be studied.
**Trial end points**	**Primary endpoints**<br><br>The primary outcome, weight regain after 18 months (T18), is defined as changes of BMI between randomization (baseline after weight loss (month 0, T0)) and T18.

	**Secondary endpoints**<br><br>The following secondary endpoints will be analysed within this trial:<br>• Effects of weight loss on hormonal counter-regulation. This will especially include hormones known to be involved in energy metabolism and satiety, e.g. thyroid hormones, glucocorticoid, insulin, incretins, leptin, growth hormone axis, estimates of sympathetic nervous activity and others.<br>• Effects of maintenance intervention on hormonal circuits. This will especially include hormones known to be involved in energy metabolism and satiety, e.g. thyroid hormones, glucocorticoid, insulin, incretins, leptin, growth hormone axis, estimates of sympathetic nervous activity and others.<br>• Effects of several anthropometric, hormonal, metabolic and behavioral parameters at T0 and weight loss induced changes of those parameters on body weight regain, metabolism and cardiovascular risk factors<br>• Effects of several anthropometric, hormonal, metabolic and behavioral parameters at T-3 on body weight regain, metabolism and cardiovascular risk factors<br>• Effects of weight loss and weight maintenance intervention on body composition<br>• Effects of weight loss and weight maintenance intervention on metabolism (HOMA-IR, ISI_{Clamp}, lipid pattern, HbA1c, blood pressure, heart rate, and others)<br>• Effects of weight loss and weight maintenance intervention on cardiovascular risk factors (lipids, blood pressure, glucose metabolism, Framingham Risk Score, carotid intima-media thickness and others)

	• Effects of weight loss and weight maintenance intervention on food pattern<br>• Effect of individual food pattern on weight maintenance<br>• Effects of weight loss and weight maintenance on tissue specific mRNA and protein expression<br>• Effects of tissue specific mRNA and protein expression at T0 and weight loss induced changes of those parameters on body weight regain, metabolism and cardiovascular risk factors<br>• Effects of tissue specific mRNA and protein expression at T-3 on body weight regain, metabolism and cardiovascular risk factors<br>• Drop out rate<br>• Interaction between chronotype and weight course<br>• Effects of age and gender
**Intervention**	Pre-trial weight loss phase: Overweight or obese subjects will participate in a standardized weight reduction program for 12 weeks to achieve a weight loss of at least 8 %.<br>12 months randomized weight maintenance phase: Subjects who lost at least 8 % of their body weight during the weight loss phase will be considered to be eligible for randomization.<br>Subjects in the control group were no longer involved in any form of counseling. Subjects in the intervention group received continuous counseling for the next twelve months.<br>Follow up period: After 12 months all subjects will undergo a free living period of six months without any further active intervention.
**Inclusion Criteria**	Inclusion Criteria:<br>• BMI > 27 kg/m2<br>• Age > 18 yrs

**Exclusion Criteria**	Exclusion Criteria:<br>• weight loss of more than 5kg in the last 2 months<br>• pregnancy, breastfeeding<br>• patients with:<br>• heart failure<br>• impaired hepatic or renal function<br>• anaemia<br>• disturbed coagulation<br>• infection, malabsorption<br>• severe hypertension<br>• any other endocrine disorder<br>• changes of smoking habits or diets within the last 3 months prior to study inclusion
**Study Duration**	18 months (after 12 week weight loss period) including a 12-month intervention period<br>30 months follow up
**Statistical methods:**	The primary outcome will be analyzed by an intention to treat (ITT) analysis (primary analysis) as well as a per-protocol analysis (secondary analysis). The intention-to-treat (ITT) population is defined by all randomized patients who have participated in at least one study visit. Missing data will be replaced by the last observed value (last observation carried forward, LOCF).<br>Secondary endpoints will be analysed as per-protocol analyses. Prediction analyses will be performed using linear or logistic regression models.
**Number of subjects**	150 male or female subjects
**Number of sites**	n=1
**Data collection**	Data collection will be performed by CRF

2 Summary

Life-style interventions show a sustained weight reduction in 10-20% of participants; however, most patients fail to maintain their weight loss. The considerable inter-individual variation of weight regain suggests that differences in the endogenous response to weight loss or environmental factors might shift the endogenous set point of body weight. Various endocrine circuits have been identified to signal information about the body energy stores to the brain, while others act as effectors integrating brain activity into behavioral and metabolic responses. Weight loss induces a coordinated response of this endocrine network favoring weight regain. The inter-individual variability and the dynamics of those hormonal circuits are unknown. Therefore, since we have the clinical expertise, we will perform a randomized, controlled, clinical trial addressing these issues in adults. After an initial 12-week run-in weight loss diet, participants will undergo a randomized 12-month, multimodal intervention trial based on physical activity and nutritional counseling. Only individuals who lose at least 8% of their initial body weight within the run-in period will be recruited to participate in the randomized weight maintenance trial. In the intervention group, an individual nutritive counseling based on regular meetings will be performed and moderate exercise will be offered; the control group will receive a brief advice leaflet, but no further intervention. Detailed metabolic phenotyping of the participants will be performed at baseline, after the 12-week run-in phase, and after the 12-month weight maintenance period. The phenotyping will include the analysis of numerous hormonal mechanisms potentially counter-balancing weight loss. Finally, the sustained effects of the intervention will be investigated by a follow-up 6 months after the end of the intervention period, which will initiate regular recalls of the participants up to 48 months under free-living conditions. This follow-up period will help us estimate, whether or not a short-term intervention is able to induce at least in some individuals, a sustained modification of the set point regulating body weight. This trial will improve the prediction of the outcome of a weight reduction and will set the basis for an in-depth understanding of the variability and the dynamics of hormonal mechanisms modifying energy homeostasis. The long-term results may help to initiate targeted and individualized therapeutic interventions aiming for body weight maintenance after weight loss. We will focus our attention on determining the difference between the weight-reducers who can sustain weight loss, compared to those who cannot.

3 Background and Rationale

Obesity is a chronic disorder and is emerging as a health problem in many developed and developing countries (18). Overweight and obesity are among the leading causes of preventable death, predominantly by inducing cardiovascular risk factors such as hypertension, dyslipidemia and diabetes mellitus. Weight loss is known to improve those risk factors and this benefit appears to persist as long as weight reduction is maintained. However, most studies indicate that long-term maintenance of weight loss is limited (19, 20). Thus, preceding weight loss and attempts of weight control are strong predictors of subsequent weight regain. Reduced body weight is usually associated with decreased energy expenditure, reflecting an orexigenic (weight gain promoting) metabolic response to the initial weight loss and suggesting a set-point of body weight, which is defended against excursion of body energy stores (21). Given that a comparable orexigenic response occurs in weight-losing obese individuals, a shifted, higher set point of body weight can be assumed in obese patients as part of the pathophysiology of obesity. The shifted, higher set point counteracts efforts to maintain reduced body weight during treatment of obesity. In recent years, our understanding of how information about body composition is linked to the brain and finally integrated into behavioral and metabolic responses has substantially improved. Endocrine circuits such as leptin, ghrelin, insulin, inkretines, thyroid hormones or sympathetic drive act coordinately to modify energy expenditure and body weight (22, 23).

Despite the apparent impact of those hormonal mechanisms counter-balancing weight loss, the inter-individual variability and the dynamics of those hormonal circuits after an intended weight loss have not yet been described. Specifically, the effect of life-style interventions is unclear. Although life-style interventions have been shown to be effective in terms of body weight reduction as long as they persist, some individuals regain weight relatively fast after such an intervention and sometimes the weight regain even exceeds the initial weight loss. However, a considerable proportion of individuals maintain a weight loss of more than 3% of the initial body weight over as long as 5 years of follow-up. This suggests that a life-style intervention may help to overcome the shifted higher set point, either by increased conscious efforts overriding the endogenous endocrine orexigenic forces or by a sustained shift of the body weight set-point to lower, normal weight levels. Although numerous studies investigated the effects of different life-style interventions, none of those aimed to comprehensively characterize the effect of such an intervention on the variability and the dynamics of hormonal mechanisms counterbalancing weight loss in a sufficiently powered study. According to existing trials, which investigated specific hormonal systems during weight reduction, the

inter-individual variation of those endocrine responses is considerable. This suggests that therapeutic approaches targeting specific endocrine circuits may offer the opportunity to support weight loss at an individualized basis. Indeed the potential success of such an approach which is based on the adjustment and normalization of an endocrine dysbalance has been demonstrated in a small, but highly standardized group of individuals (24). However, any such strategy requires a detailed and sufficiently powered analysis of those mechanisms. We therefore aim to perform a randomized controlled clinical trial addressing this topic.

4 Overview of Trial Design

4.1 Specific hypotheses and aims

The goal is to conduct a prospective randomized intervention study in obese or overweight adults. Specific aims of this proposal are:

Primary aim:

1. To investigate whether or not an intensive life style intervention during the weight maintenance period prevents body weight regain

Secondary aims:

2. To describe the inter-individual variability and the dynamic of hormonal mechanisms counter-balancing sustained weight loss.
3. To investigate whether or not an intensive life style intervention during the weight maintenance period influences the endogenous individual counter-regulation responses, thereby suggesting a modification of the individual set point of body weight.
4. To analyze the predictive impact of endocrine and metabolic factors on body weight regain

4.2 General study design

After an initial 12-week run-in weight loss diet period, participants will undergo a randomized 12-month, multimodal intervention trial based on physical activity and nutrition counseling (weight regain prevention phase).

Initial weight loss will be supported by formula very-low calorie diets. Only individuals who lose 8% of initial body weight are allowed entry into the randomized, weight maintenance trial.

In the intervention group, an individual counseling based on regular meetings will be performed. The diet will be composed according to the guidelines of the German Association of Nutrition. Dieticians will advise on weight control and reinforce the diet composition through recipes, cooking advice, and behavior modification. Moderate exercise will be performed during the last 60 min at the meetings.

The control group will receive a brief advice leaflet, but no further intervention. To estimate the long-term effects of such an intervention, a subsequent follow-up 6 months after the end of the intervention period will be performed. All participants will receive an incentive of 100 € at the end of the study to reassure compliance. The applicants intend to subsequently follow-up participants all 12 months up to month 48.

Study Flow

Screening

↓

Informed consent

↓

Inclusion in weight loss phase and phenotyping

↓

weight loss phase (3 months)

↓

Phenotyping and randomization

↓

Intervention or control group (12 months)

↓

End of intervention and phenotyping after 12 months

↓

Follow up

↓

phenotyping after 18 months

↓

long term follow up to 48 months

Figure 1. Study flow of the randomized controlled trial.

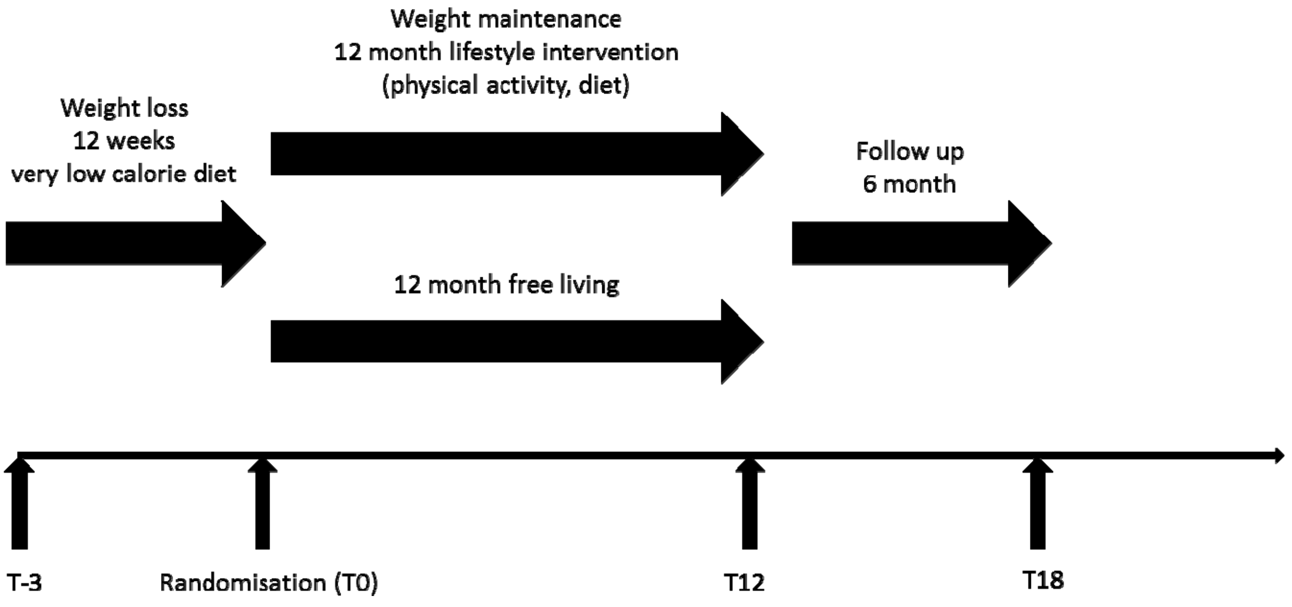

Figure 2. Time scale of the randomized controlled trial.

Time points

T-3 Start of the weight loss period

T0 Start of the randomized intervention period

T12 End of the randomized intervention period

T18 Follow-up at 18 months, 6 months after the randomized intervention period

5 Interventions

5.1 Pre-trial weight loss phase:

The protocol of the 12 weeks weight loss program will include caloric restriction, nutritional counseling and physical exercises. Therefore, weekly meetings will be performed in groups.

Caloric restriction will be applied during the weight reduction program in two stages. The first stage will based on a replacement of all three meals by a very-low energy diet (Optifast 2®, Nestlé HealthCare Nutrition GmbH, Frankfurt am Main, Germany) for eight weeks. Each participants will receive 35 portions of formula diet for each week (five per day) within the weekly meetings. All participants will be advised by the nutritionist not to consume any additional food. After eight weeks of very-low energy diet, the diet will be switched to an energy-reduced healthy diet to facilitate further weight loss. Therefore a balanced mix with

the following distribution of macronutrients: carbohydrates 35-45 %, fat 25-35 %, and protein 25-30 % as well as a daily calorie intake of approximately 1500 kcal will be recommended by the nutritionist during this phase. This will be adapted in accordance to the eating protocols, measured energy expenditure, reported physical activity and weight change in an individual approach.

This dietary approach will be supported by weekly meetings during the entire weight loss period of 12 weeks. This will also include dietary advices for healthy living and recommendation regarding increased physical activity. Within these meetings nutrition consultants will also perform group workshops with practical cooking exercises. Compliance of the diet will be supported by providing specific recipes, cooking advices, and instructions for behavior modifications. This will focus on only three meals per day, at least 4 hours break between the meals and reduced carbohydrate intake at dinner.

To further increase physical activity supervised 30 min exercise sessions will be performed after each of the weekly meetings and participants will be encouraged to attend at least one additional physical exercise course per week.

Psychologists will attended the workshops during four dates at week 5, 7, 9 and 11 and a physician will give a medical advice during one of the meetings.

5.2 12 months randomized weight maintenance phase:

Intervention group:

Subjects in the intervention group will receive continuous counseling for the next twelve months in gradually diminishing frequency. Weekly group sessions will be performed for the first 16 weeks of the 12 months study period. These will be comparable to sessions of the weight loss period. Subsequently meetings will be performed each two weeks over a period of two months. Afterwards meetings will be performed monthly until month 12. Therefore, 36 meetings will be offered to the intervention group during weight maintenance period.

Dietary advices will focus on a balanced diet within these meetings. The distribution of macronutrients will be comparable to the recommendation of the final phase of the weight loss period. Counseling will focus on dietary recommendations advocating the preferential intake of specific foods (like high intake of vegetables, cereals, fat reduced foods, lean meat consumption (lean fish and chicken)). An individual caloric intake will be calculated and nutritionist will adapt this calculation to achieve body weight maintenance. Therefore, body weight will be measured during every group session. Further weight loss will be allowed. In

case of body weight gain within this intervention period, a lower energy intake (500 kcal below the calculated energy demand) will be recommended.

In addition to the counseling, supervised physical activity regime will be maintained for the first 12 weeks of weight maintenance period. Afterwards participants will be encouraged to exercise at least twice a week but without direct supervision. The mentioned psychological support will be continued for six additional dates.

Control group:

The control group will receive a brief advice leaflet, but no further intervention. To estimate the long-term effects of such an intervention, a subsequent follow-up 6 months after the end of the intervention period will be performed.

All participants will receive an incentive of 100 € at the end of the study to reassure compliance. The applicants intend to subsequently follow-up participants all 12 months up to month 48.

6 Definition of primary and secondary endpoints

6.1 Primary endpoints

The primary outcome, weight regain after 18 months (T18), is defined as changes of BMI between randomization (baseline after weight loss (month 0, T0)) and T18.

6.2 Secondary endpoints

The following secondary endpoints will be analysed within this trial:

- Effects of weight loss on hormonal counter-regulation. This will especially include hormones known to be involved in energy metabolism and satiety, e.g. thyroid hormones, glucocorticoid, insulin, incretins, leptin, growth hormone axis, estimates of sympathetic nervous activity and others.
- Effects of maintenance intervention on hormonal circuits. This will especially include hormones known to be involved in energy metabolism and satiety, e.g. thyroid hormones, glucocorticoid, insulin, incretins, leptin, growth hormone axis, estimates of sympathetic nervous activity and others.

- Effects of several anthropometric, hormonal, metabolic and behavioral parameters at T0 and weight loss induced changes of those parameters on body weight regain, metabolism and cardiovascular risk factors
- Effects of several anthropometric, hormonal, metabolic and behavioral parameters at T-3 on body weight regain, metabolism and cardiovascular risk factors
- Effects of weight loss and weight maintenance intervention on body composition
- Effects of weight loss and weight maintenance intervention on metabolism (e.g. HOMA-IR, ISI_{Clamp}, lipid pattern, HbA1c, blood pressure, heart rate, and others)
- Effects of weight loss and weight maintenance intervention on cardiovascular risk factors (lipids, blood pressure, glucose metabolism, Framingham Risk Score, carotid intima-media thickness and others)
- Effects of weight loss and weight maintenance intervention on food pattern
- Effect of individual food pattern on weight maintenance
- Effects of weight loss and weight maintenance on tissue specific mRNA and protein expression
- Effects of tissue specific mRNA and protein expression at T0 and weight loss induced changes of those parameters on body weight regain, metabolism and cardiovascular risk factors
- Effects of tissue specific mRNA and protein expression at T-3 on body weight regain, metabolism and cardiovascular risk factors
- Drop out rate
- Interaction between chronotype and weight course
- Effects of age and gender

7 Study Population

The study will investigate the effect of a life-style intervention on body weight maintenance in overweight and obese adults.

7.1 Inclusion criteria:

- BMI > 27 kg/m2
- Age > 18 yrs

7.2 Exclusion criteria:

- weight loss of more than 5kg in the last 2 months
- pregnancy, breastfeeding
- patients with:
- heart failure
- impaired hepatic or renal function
- anaemia
- disturbed coagulation
- infection, malabsorption
- severe hypertension
- any other endocrine disorder

7.3 Recruitment

Subjects will be recruited via leaflets and the Clinic of Endocrinology, Diabetes and Nutrition.

7.4 Screening

Interested participants will be first screened by phone. The phone interview will be designed to exclude individuals who are clearly ineligible or unlikely to benefit from participation in the study. Afterward all potential eligible individuals will be screened during a clinic visit. At the screening clinic visit, medical and functional exclusions will be assessed in detail. This includes assessment of medical history and physical examination. All exclusion criteria will be evaluated. Screening will be particularly performed to rule out of abnormal thyroid function and hypercortisolism. Therefore TSH levels and 1 mg dexamethasone suppression tests will be performed. Moreover, eGFR, liver enzymes, sodium, potassium, calcium as well as lipid profile (LDL, HDL, total cholesterol and triacylglycerol) will be measured.

8 Measures and Procedures

8.1 Informed Consent

Approval from ethical committee for human studies

The study protocol was approved by the Institutional Review Board of the Charité Medical School and only subjects, who give written informed consent prior to inclusion in the study, will be included in the trial.

Approval Number: EA2/017/09
Board Name: Institutional Review Board of the Charité Medical School,
Board Affiliation: Institutional Review Board of the Charité Medical School,
Phone: 030/450-517222
Email: ethikkommission@charite.de

Address:
Dr. med. Katja Orzechowski
Charité - Universitätsmedizin Berlin
Ethikkommission
GESCHÄFTSSTELLE
Schumannstr. 20/21
10117 Berlin

Approval for animal studies

Does not apply

8.2 Procedures

8.2.1 Phenotyping

A comprehensive phenotyping will be performed before (T-3) and after (T0) weight loss and twelve months (T12) after randomization. A limited phenotyping will be performed after further six months without active intervention (T18). Afterwards this phenotyping will be repeated during follow-up all 12 months up to month 48.

Phenotyping at T-3, T0 and T12 will include:
anthropometry (bioimpedance measurements to estimate body composition, height and weight (for BMI); waist, hip and blood pressure measurements, skinfold thickness)
oral glucose tolerance test (OGTT)
24h-urine sampling (cortisol and metanephrines)
3-day nutrition protocol
questionnaires to the individual circardian rhythm (Munich Chronotype Questionnaire)
assessment of physical fitness by measuring aerobic capacity
indirect calorimetry
biopsies of adipose (abdominal subcutaneous adipose tissue samples (up to 1.0 g) by repeated needle biopsies from the periumbilical region) and muscle tissue (gastrocnemius muscle using the same approach).
bio-sampling (blood, salivia)
hyperinsulinemic euglycemic clamps
cortisone kinetics

Therefore each subject will participate in a five-day protocol. To avoid interactions between the study procedures, the phenotyping procedures will be planned and carried out at intervals of at least two days.

8.2.2 Anthropometry

Height and weight (for BMI); waist, hip and blood pressure measurements and skinfold thickness will be evaluated.

8.2.3 Bioimpedance measurements to estimate body composition

Subjects will be asked for last food and fluid intake. Body composition will be assessed in fasting state by bioelectric impedance analysis on resting participants using AKERN BIA 101 (SMT medical GmbH & Co. KG, Würzburg, Germany) after a 20 minute resting period.

8.2.4 3-day nutrition protocol

Food and fluid intake of 3 days (including 1 day at weekend) will be documented by the participants.

8.2.5 Social, Economic and Health Related Questions

For descriptive purposes, the following participant characteristics will be collected: age, gender, race, living situation, household composition, marital status, educational level, income level, smoking status, alcohol consumed, employment status. Moreover, medical history and family history will be assessed.

The following specific questionnaires will be used:
International Physical Activity Questionnaire (IPAQ)
SF-36
Eating Behavior Inventory (EBI)
Munich Chronotype Questionnaire

8.2.6 Assessment of physical fitness by measuring aerobic capacity

Physical fitness will be assessed by treadmill ergometry (h/p/cosmos para graphics mercury med 4.0; cosmos sportsmedical GmbH, Nussdorf-Traunstein; Germany). Therefore, stepwise increase of physical stress will be performed by increasing the elevation of the treadmill every two minutes. This will be done electronically controlled in steps of 3 % elevation up to the maximal exercise capacity. Therefore, Borg's scale, that ranges from 6 to 20, will be used and testing procedure will be finished at individual maximal capacity of the participants. For patient safety and stability, padded front and side rails are available and an emergency stop button is available. Heart rhythm will be controlled by continuous ECG.

8.2.7 Indirect calorimetry

Subjects will be asked for last food and fluid intake. Following a 10-hour overnight fast resting energy expenditure will estimated using indirect calorimetry via a ventilatory hood (Vmax ENCORE, CareFusion Germany 234 GmbH, Germany) after a 20 minute resting period at 8.00 a.m.

8.2.8 Oral glucose tolerance test (OGTT)

Subjects will be asked for last food and fluid intake. Oral glucose tolerance test with 75 g glucose will performed in fasting state and blood samples will be taken at 0, 30, 60, 90, 120

and 180 min. Blood samples will centrifuged, and plasma and serum samples will be frozen immediately at -80°C.

8.2.9 Biopsies of adipose and muscle tissue

Biopsies will be taken at T-3, T0 and T12 from different body sides. Abdominal subcutaneous adipose tissue samples (0.5 to 1.0 g) will be taken by repeated needle biopsies from the periumbilical region using a 12 G biopty-cut needle (CR Bard GmbH, Karlsruhe, Germany). Muscle biopsies will be taken from the gastrocnemius muscle using the same approach. At first, the skin will be anesthetized with 1% lidocaine without epinephrine. Subsequently, a skin incision (3-4 mm) will be performed and biopsies will be taken using a biopty-cut needle (CR Bard GmbH, Karlsruhe, Germany). Both fat and muscle samples must snap-frozen in liquid nitrogen and be stored at -80°C until further analysis.

8.2.10 Hyperinsulinemic euglycemic clamps

A stable infusion of 40 $mIU•m^{-2}•min^{-1}$ human insulin (Actrapid®, Novo Nordisk, Bagsvaard, Denmark) and a variable infusion of 10 % glucose (Serag Wiessner, Naila, Germany) will be used to maintain blood glucose between 4.0 and 4.9 mmol/l. Therefore, capillary glucose concentration will be monitored every 5 minutes and the glucose infusion rate will be varied appropriately. Blood samples will be collected before the clamp and at least two hours after starting the clamp during steady-state conditions. Steady state is defined by blood glucose between 4.0 and 4.9 mmol/l for at least 30 minutes with only minimal changes of glucose infusion rate. Moreover, steady state must be at least two hours after starting the clamp.
Blood samples will centrifuged, and plasma and serum samples will be frozen immediately at -80°C.

8.2.11 Cortisone kinetics

Conversion of cortisone to cortisol by 11β-HSD1 on first pass through the liver will be measured by cortisone kinetics. To suppress endogenous cortisol production on this day, 1 mg oral dexamethasone will be administered at 24.00 h and 0·5 mg dexamethasone at 06.00 h after fasting overnight. Blood will be taken at 08.00 h. Afterwards 37.5 mg cortisone acetate will be given orally and blood samples for measurement of serum cortisol and cortisone will be collected every 30 min for 300 min.

8.2.12 Saliva

Saliva sample will be collected at 8.00, 12.00, 16.00, 20.00 und 24.00 h.

8.2.13 24h-urine sampling (cortisol and metanephrines)

24-hours urine sampling will be performed in each subject on two independent days.

8.2.14 Intima media thickness

Intima media thickness (IMT) will be measured at both carotid arteries in the supine position with the head tilted backwards using a high-resolution ultrasound (Kretz Soluson 730; Kretz Technik, Marl, Germany). Carotid arterial IMT will be analyzed at the posterior wall of the common carotid artery and the bulbus at three different positions and mean values of those measurements will be calculated.

8.3 Assessment Schedule

The schedule of clinic visits, procedures and assessments is summarized in Table 1

Table 1: Assessments schedule

**Visit day**	**T-3**	**T0**	**T12**	**T18**
**Questionnaires**	✓	✓	✓	✓
**Health status and SAE**	✓	✓	✓	✓
**Medication**	✓	✓	✓	✓
**Anthropometry**	✓	✓	✓	✓
**Physical examination**	✓	✓	✓	✓
**Blood sampling**	✓	✓	✓	✓
**OGTT**	✓	✓	✓	✓
**hyperinsulinemic euglycemic clamps**	✓	✓	✓	
**Biopsies**	✓	✓	✓	
**24h-urine sampling**	✓	✓	✓	
**Cortisone kinetics**	✓	✓	✓	
**Salavia**	✓	✓	✓	
**Indirect calorimetry**	✓	✓	✓	✓
**Intima media thickness**	✓	✓	✓	
**Physical fitness**	✓	✓		
**3-day nutrition protocol**	✓	✓	✓	✓

8.4 Randomization

A stratified randomization of the participants will be performed using a randomization list. Stratification will consider gender and body weight at baseline (3 BMI strata).

8.5 Participant Safety

Risks associated with a weight reduction will be assessed. Therefore fat metabolism (i.e. increase of triglycerides), renal function and electrolytes will be analyzed at baseline, after 4 and 8 weeks during the initial phase of the very low calorie diets at frequent intervals. Body weight will be measured weekly during the 12-week weight loss period. In the first two months weight loss and health status will be monitored weekly, which included a patient interview and blood pressure measurement. Body weight will be measured at least once per week. A Data Safety Monitoring Board (DSMB) will be established, with responsibility to monitor all safety aspects of the study. The Medical Safety Committee reports to the DSMB for issues related to participants safety. The monitoring will be provided by the KKS of the Charité.

A subject will be excluded from the trial if:

- the user's own request (also without a reason)
- this is considered necessary by the investigator or head of the study from a medical point of view (e.g., due to abnormal laboratory values or pregnancy)
- the subject does not follow the study protocol (substantial deviation from the recommended diet).
- severe disease
- inclusion criteria are no longer met
- new scientific findings during the study's lifetime, which are against the continuation of the clinical trial

9 Data management

Data collection will be performed using CRF. All data will be transferred by independent staff into an electronic data base.

10 Statistical Considerations

10.1 Power analysis and sample size calculation

Weight regain will be used for power calculation. This considers the fact that the variance of any causal endocrine parameter should be smaller than that of body weight, since the latter is also affected by many other parameters such as socio-economic or life-style factors, which do not modify endocrine circuits. Previous studies in adults and children have demonstrated that a lifestyle intervention after an initial weight loss results basically in a successful weight maintenance over at least 6 months, while a substantial regain of weight was found in the control group (i.e. 3.3 ± 0.74% of the body weight after initial weight reduction) [1-4]. The here performed power calculation considered the subsequent follow-up after 6 months without intervention (T18). An α- and β- error rate of 5 and 20 % was considered, respectively. Assuming a reduction of the effect size by about 30% compared to T12 and given a variance of 1.96% in the control group, the power calculation resulted in 46 individuals per treatment arm (query 7.0). Assuming a 20% drop out rate during the initial weight loss period and about 15% drop outs during the randomized intervention a total of at least 144 individuals had to be included in the weight reduction period (T-3).

10.2 Data analysis plan

The primary outcome will be analyzed by an intention to treat (ITT) analysis (primary analysis) as well as a per-protocol analysis (secondary analysis). The intention-to-treat (ITT) population is defined by all randomized patients who have participated in at least one study visit.

Mixed-model, repeated-measures analysis of variance will be used to analyze primary outcome. This will consider the correlation between repeated observations and will use all available subsequent observations for all participants with values at randomization, regardless of further assessment completion. Means will be modeled as a function of group assignment and study visit (T0 and after 12 (T12) and 18 months (T18)). Adjustment for gender, age and BMI before weight loss will be included in this model.

Secondary endpoints will be analysed as per-protocol analyses. This will include mixed-model, repeated-measures analysis of variance to compare hormonal levels of intervention and control group within the trial. Adjustment for potential confounders like gender, age and BMI will be included in these models.

Prediction analyses will be performed using linear or logistic regression models. Effect of age, gender, BMI after weight loss (at T0) and the randomization group are predefined confounders, which will be included in these models.

11 Appendix

Questionnaires and study documents
